# Flower-Like Plasma Cell: A Comment

**DOI:** 10.4274/tjh.galenos.2021.2021.0230

**Published:** 2021-08-25

**Authors:** Smeeta Gajendra

**Affiliations:** 1 Laboratory Oncology Unit, Dr. B.R.A. IRCH, AIIMS, New Delhi, India

**Keywords:** Flower cells, Plasma cell myeloma, Flowcytometric immunophenotyping, Immunohistochemistry

## To the Editor,

I read “Flower-Like Plasma Cell Nuclei in Multiple Myeloma” by Sall et al. [1], recently published in this journal. This image report is very descriptive and informative regarding a case of multiple myeloma showing abnormal plasma cells with flower-shaped nuclear features. These morphological features can pose a diagnostic dilemma and can mimic lymphoma as “flower cells” or clover-leaf lymphocytes are described typically in HTLV-1-induced adult T-cell leukemia and very rarely in B-cell lymphoma [2]. Plasma cell myeloma or leukemia rarely presents with flower‐shaped nuclei and occasional cases of plasma cell leukemia mimicking adult T-cell leukemia/lymphoma were previously reported in the literature [3,4]. Upon flowcytometric immunophenotyping, the absence of B-cell or T-cell markers and the presence of plasma cell markers with strong CD38 and CD138 help in differentiating it from lymphoma. The morphological variation of abnormal plasma cells in plasma cell neoplasms is vast, ranging from small lymphocyte-like cells to cleaved, convoluted, monocytoid or multilobated plasma cells to anaplastic pleomorphic large plasma cells, which have been reported previously. A few cases of morphological variants of plasma cell neoplasms, like megakaryocytic, plasmoblastic, or megakaryoblastic mimicking acute leukemia, have also been reported in the literature [5]. The presence of cytoplasmic granulations, vacuolations, crystals (mimicking histiocytes), Auer rod-like inclusions, or cytoplasmic projections has also been noted in the literature. Morphological transformation of plasma cells into multilobated nuclei during the clinical course followed by anaplastic myeloma transformation is also occasionally reported [6]. Circulating cells with cleaved, multilobated, or monocytoid nuclei can be present in a variety of non-hematologic and hematologic disorders, such as reactive plasmacytosis associated with breast carcinoma, metastatic carcinoma, plasma cell leukemia, myelomonocytic leukemia, malignant lymphoma, and multiple myeloma [7]. Autoimmune disorders, hepatitis C, human immunodeficiency virus infections, angioimmunoblastic T-cell lymphoma, and Hodgkin lymphoma are a few examples in which reactive plasmacytosis of bone marrow may reach levels of up to 30%-50%. Abnormal plasma cells can be differentiated from normal or reactive plasma cells on flowcytometric immunophenotyping as abnormal plasma cells are mostly CD19-, CD20+, CD27-, CD28+, CD117+, CD56+, CD33+, CD200++, CD307++, CD81-weak to -negative, and clonal for kappa or lambda immunoglobulin [8]. The presence of flower cells in this case demonstrates the use of immunophenotyping and FISH/cytogenetic studies in the classification of atypical, multilobated flower-shaped mononuclear cells and also that flower cell morphology is not restricted only to lymphomas. Sometimes neoplastic plasma cells exhibit cytoplasmic heterogeneity, which poses difficulty in morphological diagnosis and requires ancillary technology like biopsy with immunohistochemistry or immunophenotyping for a definitive diagnosis.
