# Supplementary material for: Flower-Like Plasma Cell: A Comment
Source: Turk J Haematol. 2021 Aug 25;38(3):228–9. doi: 10.4274/tjh.galenos.2021.2021.0230 (PMC8386317; doi:10.4274/tjh.galenos.2021.2021.0230)
Supplement: Related texts

# REPLY FROM THE AUTHORS

We thank Dr. Gajendra for the interest shown in our article. She described in detail the morphological abnormalities, both nuclear and cytoplasmic, of tumoral plasma cells as well as reactive plasma cells.

We fully agree with her that, when in doubt, immunophenotyping is an indispensable tool for distinguishing plasma cells.

Unfortunately, we do not have the cytogenetics to deepen this characterization.

Regards,

**Abibatou Sall, Moussa Seck, Diama Samb, Blaise Félix Faye, Macoura Gadji, Saliou Diop, Awa Oumar Touré**
